# Supplementary material for: Inhibition of Pseudomonas aeruginosa quorum sensing by chemical induction of the MexEF-oprN efflux pump
Source: Antimicrob Agents Chemother. 2024 Jan 8;68(2):e01387-23. doi: 10.1128/aac.01387-23 (PMC10848761; doi:10.1128/aac.01387-23)
Supplement: Supplemental figures and tables and synthetic procedures — Tables S1 to S8 and Figures S1 to S8. [file aac.01387-23-s0001.pdf]

Supplemental Material

Inhibition of *Pseudomonas aeruginosa* quorum sensing by chemical induction of the *mexEF-oprN* efflux pump

Rasmus Kristensen[a], Jens Bo Andersen[a], Morten Rybtke[a], Charlotte Uldahl Jansen[b], Blaine Gabriel Fritz[a], Rikke Overgaard Kiilerich[a], Jesper Uhd[b], Thomas Bjarnsholt[a,c], Katrine Qvortrup[b], Tim Tolker-Nielsen[a], Michael Givskov[a] and Tim Holm Jakobsen[a*]

[a]Costerton Biofilm Center, Department of Immunology and Microbiology, University of Copenhagen, DK-2200 Copenhagen, Denmark.

[b]Department of Chemistry, Technical University of Denmark, DK- 2800 Kgs. Lyngby, Denmark.

[c]Department of Clinical Microbiology, Rigshospitalet, DK-2100 Copenhagen, Denmark.

Supplemental tables

Table S1 Strain and plasmid list.

Strain or plasmid	Description	References
P. aeruginosa		
PAO1	Wild type *P. aeruginosa*	[1]
rhlA-gfp	PAO1 carrying plasmid pMHRA encoding a P_{rhlA}-gfp(ASV) fusion, Gm^R	[2]
lasB-gfp	PAO1 carrying one copy of the P_{lasB}-gfp (ASV)::P_{lac}-lasR expression cassette of pTn5-LAS on its chromosom, Gm^R	[3]
pqsA-gfp	PAO1 carrying plasmid pAC37 encoding a P_{pqsA}-gfp(ASV) fusion, Gm^R	[4]
rsmY-gfp	PAO1 carrying plasmid pRV60_1 encoding a rsmY-gfpmut3* transcriptional fusion, Gm^R, Ap^R	[2]
rsmZ-gfp	PAO1 carrying plasmid pRV59_1 encoding a rsmZ-gfpmut3* transcriptional fusion, Gm^R, Ap^R	[2]
ΔrlhA	PAO1 *rhlA* mutant	[3]
ΔlasIΔrhlI	PAO1 *lasI-rhlI* mutant	[4]
CTX-lasB-gfp	PAO1 carrying one copy of the P_{lasB}-*gfp*(ASV)::P_{lac}-*lasR* expression cassette at the chromosomal CTX insertion site.	This study
ΔmexS	PAO1 mexS mutant	This study
ΔmexT	PAO1 mexT mutant	This study
ΔmexE	PAO1 mexE mutant	This study
ΔmexS lasB-gfp	PAO1 mexS mutant carrying one copy of the P_{lasB}-*gfp*(ASV)::P_{lac}-*lasR* expression cassette at the chromosomal CTX insertion site	This study
ΔmexT lasB-gfp	PAO1 mexT mutant carrying one copy of the P_{lasB}-*gfp*(ASV)::P_{lac}-*lasR* expression cassette at the chromosomal CTX insertion site	This study
ΔmexE lasB-gfp	PAO1 mexE mutant carrying one copy of the P_{lasB}-*gfp*(ASV)::P_{lac}-*lasR* expression cassette at the chromosomal CTX insertion site	This study
E. coli		
MS690	MG1655 ΔhsdR; cloning strain	Mark A. Schembri
DH5α	Standard cloning strain	Lab collection
S17pir	Donor strain for introduction of pBT20 during transposon mutagenesis	Lab collection
RK600	Helper strain in three-parental mating	Lab collection
**Plasmids**		
pENTRmexS-KO	ΔmexS allelic exchange vector, Gm^R	This study
pENTRmexE-KO	ΔmexE allelic exchange vector, Gm^R	This study
pENTRmexT-KO	ΔmexT allelic exchange vector, Gm^R	This study
pDONRPEX18Gm	Gateway-compatibel donor vector for creating the allelic exchange vectors	[5]
mini-CTX2	Integration vector for insertion into the chromosomal *φ CTX* site of *P. aeruginosa*., Tc^R	[6]
pMHLAS	Shuttle vector encoding a P_{lasB}-*gfp*(ASV)::P_{lac}-*lasR* expression cassette, Gm^R, Ap^R	[3]
pRK2	P_{lasB}-*gfp*(ASV)::P_{lac}-*lasR* expression cassette of pMHLAS inserted into the *NotI* site of the chromosomal integration vector mini-CTX2, Tc^R	This study
pBT20	Mariner transposon delivery vector	[7]

Table S2 Primer list

Name	Sequence 5´-> 3´	References
lasB		
Forward	CGACAACGCGTCGCAGTA	[8]
Reverse	AGGTAGAACGCACGGTTGTACA	[8]
rhlA		
Forward	GGCGATCGGCCATCT	[8]
Reverse	AGCGAAGCCATGTGCTGAT	[8]
pqsA		
Forward	CATCTCGCCGAACAGATTCC	This study
Reverse	CCAACTTGCCGTTGTCGTT	This study
rpoD		
Forward	ACAAGATCCGCAAGGTACTGAAG	[8]
Reverse	CGCCCAGGTGCGAATC	[8]
mexS		
Forward	TGA TCG CTT CCA CCA AGT CC	This study
Reverse	GAT GAC ATT GAC GCC CTT GC	This study
mexT		
Forward	TAG CCC CTG GAA ACG AGG AA	This study
Reverse	GGT CTC GAA CAC GAT CAG CA	This study
mexE		
Forward	TGA TCA AGG ACG AAG CGG TC	This study
Reverse	TGC GGT AGA CGG TCT TGT TG	This study
mexF		
Forward	TTC ATC ACC GCC GAA CTC AA	This study
Reverse	TCG GTC CAC TCG TAG GTC AT	This study
***mexS* knockout vector**		
mexS_UpF_attB1	GGGGACAAGTTTGTACAAAAAAGCAGGCTCAgcgaagatttcctgggctc	This study
mexS_UpR	CTCAATCGGCGACGTGGATtcgggacatcgcaaacctc	This study
mexS_DnF	Atccacgtcgccgattgag	This study
mexS_DnR_attB2	GGGGACCACTTTGTACAAGAAAGCTGGGTAacaacccaagcgatcaacttc	This study
***mexT* knockout vector**		
mexT_UpF_attB1	GGGGACAAGTTTGTACAAAAAAGCAGGCTCAacgccagagcacatccttc	This study
mexT_UpR	GGAGGGCTCAGAGACTgtcgtttcggttcatggcg	This study
mexT_DnF	Gacagtctctgagccctcc	This study
mexT_DnR_attB2	GGGGACCACTTTGTACAAGAAAGCTGGGTAcactcgttcagcggttgttc	This study
***mexE* knockout vector**		
mexE_UpF_attB1	GGGGACAAGTTTGTACAAAAAAGCAGGCTCAgacatcatcgccaccgtg	This study
mexE_UpR	GTTGCGAGTGGCGTTGTCctgttccatgcttgactccg	This study
mexE_DnF	Gacaacgccactcgcaac	This study
mexE_DnR_attB2	GGGGACCACTTTGTACAAGAAAGCTGGGTAgcatgtcgtagcggttatcc	This study
**Transposon mutagenesis PCR Round 1**		
Rnd1-ARB1-Pa	GGCCACGCGTCGACTAGTACNNNNNNNNNNAGAG	This study
Rnd1-ARB2-Pa	GGCCACGCGTCGACTAGTACNNNNNNNNNNACGCC	This study
Rnd1-TnM20	TATAATGTGTGGAATTGTGAGCGG	This study
**Transposon mutagenesis PCR Round 2**		
Rnd2-ARB	GGCCACGCGTCGACTAGTAC	This study
Rnd2-TnM20	ACAGGAAACAGGACTCTAGAGG	This study
**Sequencing**		
BT20TnMSeq	CACCCAGCTTTCTTGTACAC	This study

Table S2 The 50 most downregulated genes by 100 µM Z-ethylthio enynone[a]

	Downregulated			
**Gene id**	**Gene name**		**Fold change**	**Adjusted P-value**
PA2570	*lecA*	*	-157	4,19E-86
PA2069	probable carbamoyl transferase	*	-111	5,12E-201
PA2300	chiC (chitinase)	*	-86	-
PA1914	conserved hypothetical protein	*	-79	1,26E-172
PA1900	*phzB2*		-78	1,39E-139
PA3724	*lasB*	*	-77	7,97E-197
PA2068	probable major facilitator superfamily (MFS) transporter	*	-74	4,62E-190
PA4141	hypothetical protein	*	-53	3,66E-94
PA1874	hypothetical protein	*	-47	5,64E-217
PA1901	*phzC2*	*	-46	3,04E-71
PA1899	*phzA2*		-39	5,15E-35
PA0122	rahU(aegerolysin)	*	-38	8,63E-140
PA4306	*flp*		-35	1,01E-133
PA0880	probable ring-cleaving dioxygenase		-33	8,54E-45
PA0852	cbpD(chitin-binding protein CbpD precursor)	*	-33	3,93E-281
PA1871	*lasA*	*	-33	2,86E-184
PA1216	hypothetical protein		-32	9,45E-119
PA1875	*oplM* probable outer membrane protein precursor	*	-30	7,20E-148
PA0881	hypothetical protein		-30	2,94E-110
PA1215	hypothetical protein		-28	3,52E-141
PA4211	*phezB1*	*	-26	4,70E-92
PA2134	hypothetical protein		-25	1,52E-111
PA0883	probable acyl-CoA lyase beta chain		-25	1,78E-111
PA2939	pepB probable aminopeptidase*	*	-24	8,86E-143
PA0882	hypothetical protein		-24	9,73E-93
PA0878	hypothetical protein		-22	6,58E-46
PA2171	hypothetical protein	*	-22	2,01E-58
PA4651	*cupE4*		-22	1,03E-191
PA3361	*lecB*	*	-22	4,41E-75
PA4648	*cupE1*		-21	4,56E-69
PA1217	probable 2-isopropylmalate synthase		-21	7,23E-129
PA5220	hypothetical protein	*	-21	4,40E-132
PA4212	*phzC1*		-21	1,14E-75
PA1221	hypothetical protein		-19	7,18E-44
PA1220	hypothetical protein		-18	1,03E-54
PA4299	*tadD*		-18	2,92E-96
PA1214	hypothetical protein		-18	9,43E-76
PA0123	probable transcriptional regulator		-18	3,17E-98
PA3479	*rhlA*	*	-18	1,34E-58
PA4078	probable nonribosomal peptide synthetase	*	-18	3,86E-110
PA2170	hypothetical protein	*	-18	2,46E-71
PA1324,1	hypothetical protein	*	-17	7,59E-05
PA4298	hypothetical protein		-17	3,94E-61
PA1877	probable secretion protein		-16	9,27E-108
PA4209	*phzM*	*	-16	1,09E-83
PA2172	hypothetical protein		-16	1,03E-98
PA4590	pra protein activator		-16	2,68E-118
PA2169	hypothetical protein		-16	2,39E-90
PA4302	*tadA*		-16	2,85E-98
PA2166	hypothetical protein*	*	-16	2,85E-61

[a]The asterisk indicates genes previously identified as QS regulated by Hentzer et al., [9]. The results are based on three independent experiements.

Table S3 The 50 most upregulated genes by 100 µM Z-ethylthio enynone[a]

		Upregulated		
**Gene id**	**Gene name**		**Fold change**	**Adjusted P-value**
PA4623	hypothetical protein		1076	9,24E-270
PA4881	hypothetical protein		454	3,35E-300
PA4832	probable short-chain dehydrogenase		303	-
PA2493	*mexE*		149	4,94E-217
PA3229	hypothetical protein		125	5,74E-262
PA1240	probable enoyl-CoA hydratase/isomerase		117	-
PA2758	probable transcriptional regulator		111	3,56E-217
PA1942	hypothetical protein		96	3,23E-94
PA2494	*mexF*		83	5,56E-165
PA3133	*sawR*		75	2,34E-189
PA2845	hypothetical protein		68	1,59E-61
PA2759	hypothetical protein		65	8,62E-140
PA2495	*oprN*		59	1,81E-263
PA1239	hypothetical protein		57	1,14E-276
PA3132	probable hydrolase		49	4,75E-226
PA2487	hypothetical protein		45	5,70E-78
PA2486	*ptrC* (Pseudomonas type III repressor gene C)	*	41	8,75E-105
PA4830	hypothetical protein	*	39	7,20E-177
PA4831	probable transcriptional regulator		38	2,89E-118
PA1744	hypothetical protein		37	6,86E-53
PA3230	conserved hypothetical protein		36	1,13E-230
PA1743	hypothetical protein		33	3,38E-52
PA2932	*morB* (morphinone reductase)		33	7,51E-130
PA1970	hypothetical protein		29	7,45E-78
PA3450	*isfA* (1-Cys peroxiredoxin LsfA)		27	6,27E-52
PA2940	probable acyl-CoA thiolase		26	1,50E-93
PA4622	probable major facilitator superfamily (MFS) transporter		25	1,13E-86
PA0474	hypothetical protein		25	7,22E-52
PA2933	probable major facilitator superfamily (MFS) transporter		22	3,81E-51
PA2204	probable binding protein component of ABC transporter		22	8,04E-49
PA1333	hypothetical protein		20	4,40E-117
PA4023	*eutP* (probable transport protein)		20	5,31E-34
PA3444	*ssuD* (conserved hypothetical protein)		20	2,52E-71
PA2062	probable pyridoxal-phosphate dependent enzyme		18	9,42E-83
PA2935	probable pyridoxal-phosphate dependent enzyme		18	1,04E-10
PA2490	conserved hypothetical protein		17	2,77E-81
PA2934	*cif* CFTR inhibitory factor		16	5,56E-46
PA2203	probable amino acid permease		16	2,58E-17
PA2087	hypothetical protein		15	2,59E-45
PA4882	hypothetical protein		14	2,66E-50
PA2491	*mexS*		14	1,18E-113
PA1332	hypothetical protein		14	6,03E-57
PA3445	conserved hypothetical protein		14	5,73E-43
PA3443	*ycbM* probable permease of ABC transporter		14	1,46E-39
PA1374	hypothetical protein		14	1,51E-80
PA2086	probable epoxide hydrolase		13	1,15E-47
PA0442	hypothetical protein		13	0,03
PA2714	probable molybdopterin oxidoreductase		13	1,28E-96
PA2767	probable enoyl-CoA hydratase/isomerase		13	2,73E-41
PA3937	*tauB*		13	2,89E-36

[a]The asterisk indicates genes previously identified as QS regulated by Hentzer et al., [9]. The results are based on three independent experiements.

Table S4 Alterations in gene expression of the tad locus by treatment with 100 µM Z-ethylthio enynone.

tad (tight adhension) loci			
**Gene id**	**Gene name**	**Fold change**	**Adjusted P-value**
PA4306	*flp*	-35,5	5,52E-136
PA4305	*rcpC*	-9,4	2,10E-48
PA4304	*rcpA*	-13,1	9,83E-80
PA4303	*tadZ*	-14,1	4,57E-92
PA4302	*tadA*	-15,8	2,85E-98
PA4301	*tadB*	-14,0	7,47E-61
PA4300	*tadC*	-14,5	2,92E-96
PA4299	*tadD*	-18,4	3,64E-98
PA4298	hypothetical protein	-16,6	3,94E-61
PA4297	*tadG*	-4,4	1,45E-40
PA4296	*pprB*	-4,9	6,05E-33
PA4295	*fppA*	-2,3	0,01
PA4294	hypothetical protein	-8,8	2,40E-25
PA4293	*pprA*	-7,0	7,90E-31

Table S5 Alterations in gene expression of cupA-C,E gene clusters by treatment with 100 µM Z-ethylthio enynone.

cupA gene cluster			
**Gene id**	**Gene name**	**Fold change**	**Adjusted P-value**
PA2127	*cgrA*	-2,2	2,23E-07
PA2128	*cupA1*	-1,3	0,04
PA2129	*cupA2*	1,1	0,87
PA2130	*cupA3*	1,2	0,25
PA2131	*cupA4*	1,1	0,69
PA2132	*cupA5*	1,3	0,58

cupB gene cluster			
**Gene id**	**Gene name**	**Fold change**	**Adjusted P-value**
PA4087	*moaF*	1,6	0,06
PA4086	*cupB1*	-1,0	0,88
PA4085	*cupB2*	1,2	0,42
PA4084	*cupB3*	-1,7	2,16E-05
PA4083	*cupB4*	1,6	0,16
PA4082	*cupB5*	1,4	0,01
PA4081	*cupB6*	1,5	0,00

cupC gene cluster			
**Gene id**	**Gene name**	**Fold change**	**Adjusted P-value**
PA0991	*vgrG1*	1,0	0,91
PA0992	*cupC1*	-1,0	0,95
PA0993	*cupC2*	1,2	0,65
PA0994	*cupC3*	2,2	6,43E-09

cupE gene cluster			
**Gene id**	**Gene name**	**Fold change**	**Adjusted P-value**
PA4647	*uraA*	-1,9	3,18E-12
PA4648	*cupE1*	-21,5	4,56E-69
PA4649	*cupE2*	-8,7	1,32E-42
PA4650	*cupE3*	-8,3	3,97E-36
PA4651	*cupE4*	-22,3	1,03E-191
PA4652	*cupE5*	-4,9	5,72E-59
PA4653	*cupE6*	-3,7	1,33E-23

Table S6 Alterations in gene expression of the three (H1-H3) Type 6 secretion systems (T6SS) by 100 µM Z-ethylthio enynone.

		H1-T6SS	
**Gene id**	**Gene name**	**Fold change**	**Adjusted P-value**
PA0082	*tssA1*	-1,5	5,76E-04
PA0083	*tssB1*	-2,3	1,25E-07
PA0084	*tssC1*	-2,3	1,13E-06
PA0085	*hcp1*	-2,5	1,73E-04
PA0086	*tagJ1*	-1,2	0,27
PA0087	*tssE1*	1,1	0,81
PA0088	*tssF1*	-1,8	0,01
PA0089	*tssG1*	-1,8	0,02
PA0090	*clpV1*	-1,9	0,02
PA0091	*vgrG1*	-1,3	0,23
PA0092	*tsi6*	-1,1	0,51
PA0093	*tse6*	-1,1	0,51
PA0094	*eagT6*	-1,3	0,23

		H2-T6SS	
**Gene id**	**Gene name**	**Fold change**	**Adjusted P-value**
PA1656	*hsiA2*	-4,6	1,48E-11
PA1657	*hsiB2*	-6,3	5,73E-14
PA1658	*hsiC2*	-6,3	1,76E-20
PA1659	*hsiF2*	-9,2	1,07E-16
PA1660	*hsiG2*	-3,5	1,21E-15
PA1661	*hsiH2*	-3,3	1,01E-07
PA1662	*clpV2*	-3,4	2,10E-09
PA1663	*sfa2*	-3,5	2,74E-07
PA1664	*orfX*	-9,4	2,02E-04
PA1665	*fha2*	-4,3	5,73E-11
PA1666	*lip2*	-3,5	4,23E-07
PA1667	*hsiJ2*	-2,4	4,19E-06
PA1668	*dotU2*	-3,1	1,22E-05
PA1669	*icmF2*	-2,4	4,22E-06
PA1670	*stp1*	-1,9	0,01
PA1671	*stk1*	1,8	4,42E-05

		H3-T6SS	
**Gene id**	**Gene name**	**Fold change**	**Adjusted P-value**
PA2364	*lip3*	-1,4	0,15
PA2363	*hsiJ3*	-2,6	6,14E-16
PA2362	*dotU3*	-3,0	2,05E-14
PA2361	*icmF3*	-3,0	2,13E-13
PA2360	*hsiA3*	-4,2	5,77E-13
PA2359	*sfa3*	5,3	1,11E-23
PA2365	*hsiB3*	-6,8	4,97E-67
PA2366	*hsiC3*	-5,6	1,93E-48

PA2367	*hcp3*	-7,4	1,27E-54
PA2368	*hsiF3*	-5,9	1,10E-45
PA2369	*hsiG3*	-5,5	2,09E-37
PA2370	*hsiH3*	-5,3	1,63E-20
PA2371	*clpV3*	-4,8	3,41E-21
PA2372	*vgrG3*	-4,2	2,65E-10

Table S7 Observed upregulated genes in response to MexT stimulation [10] and fold change of the same genes in response to treatment with 400 µM iberin [11] and 100 µM Z-ethylthio enynone.

**Fetar et al., [10]**	**Jakobsen et al., [11] 400 µM iberin**	**This study 100 µM Z-ethylthio enynone**
	**Fold change**	
PA1744	80	37
PA1970	100	29
PA2486	95	41
PA2759	265	65
PA2811	11	6
PA2812	10	7
PA2813	23	11
PA3229	213	124
xenB	20	9
PA4623	132	1076
PA4881	746	453

Table S8 Alteration of gene expression of *mexS, mexT* and *mexEF-oprN* in response to treatment with 400 µM iberin [11] and 100 µM Z-ethylthio enynone.

		**Jakobsen et al., [11] 400 µM iberin**	**This study 100 µM Z-ethylthio enynone**
**Gene id**	**Gene name**	**Fold change**	
PA2491	*mexS*	24	14
PA2492	*mexT*	2	1
PA2493	*mexE*	100	149
PA2494	*mexF*	51	83
PA2495	*oprN*	13	59

Supplemental figures

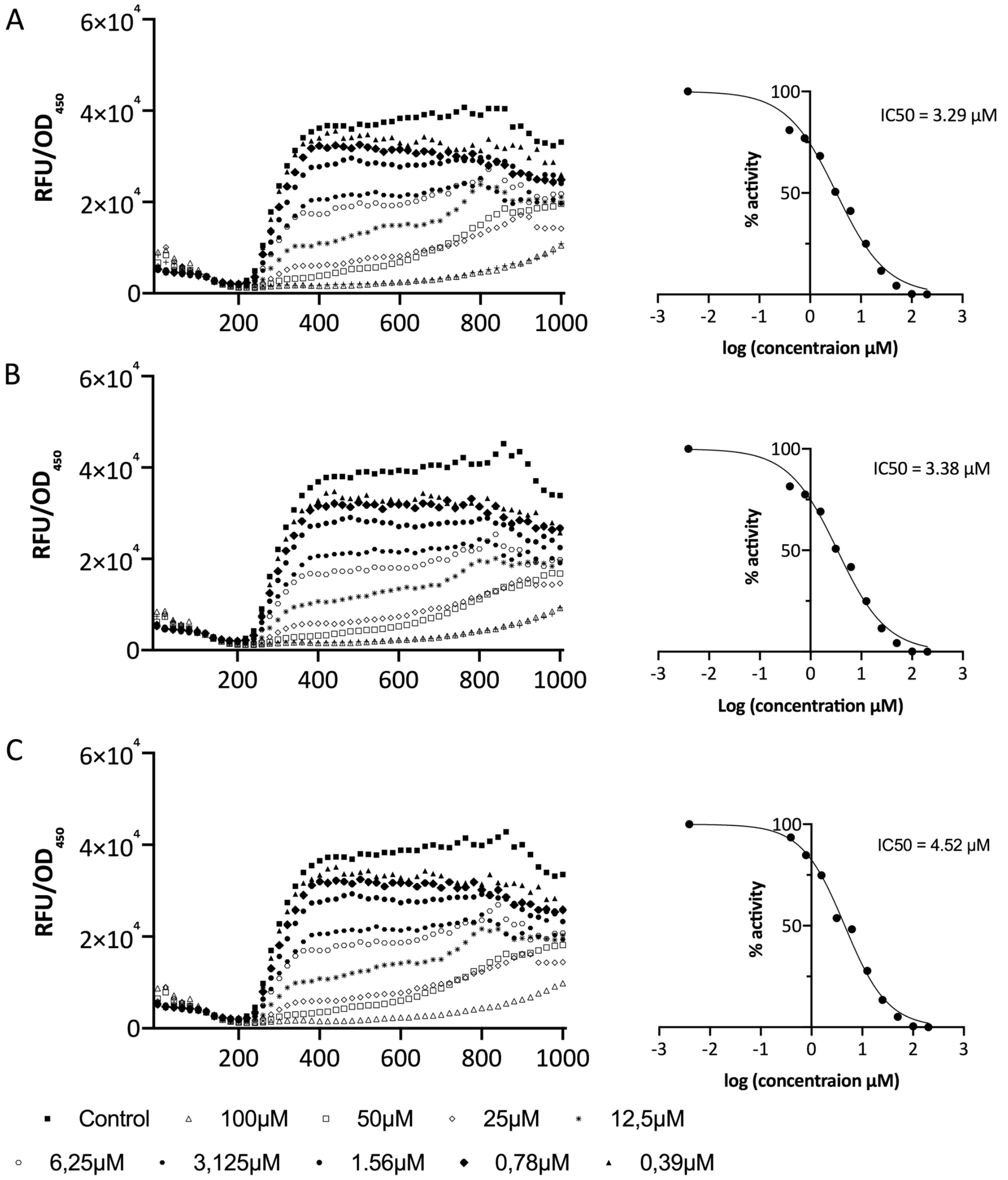

FIG S1 Inhibitory activity of Z/E-ethylthio enynone, Z-ethylthio enynone and Z/E-propylthio enynone on *lasB* expression. Expression of *lasB* measured as GFP intensity of a *lasB-gfp* strain treated with 100 µM Z/E-ethylthio enynone (a), Z-ethylthio enynone (b) and Z/E-propylthio enynone (C). With corresponding curves for calculation of IC_{50} values.

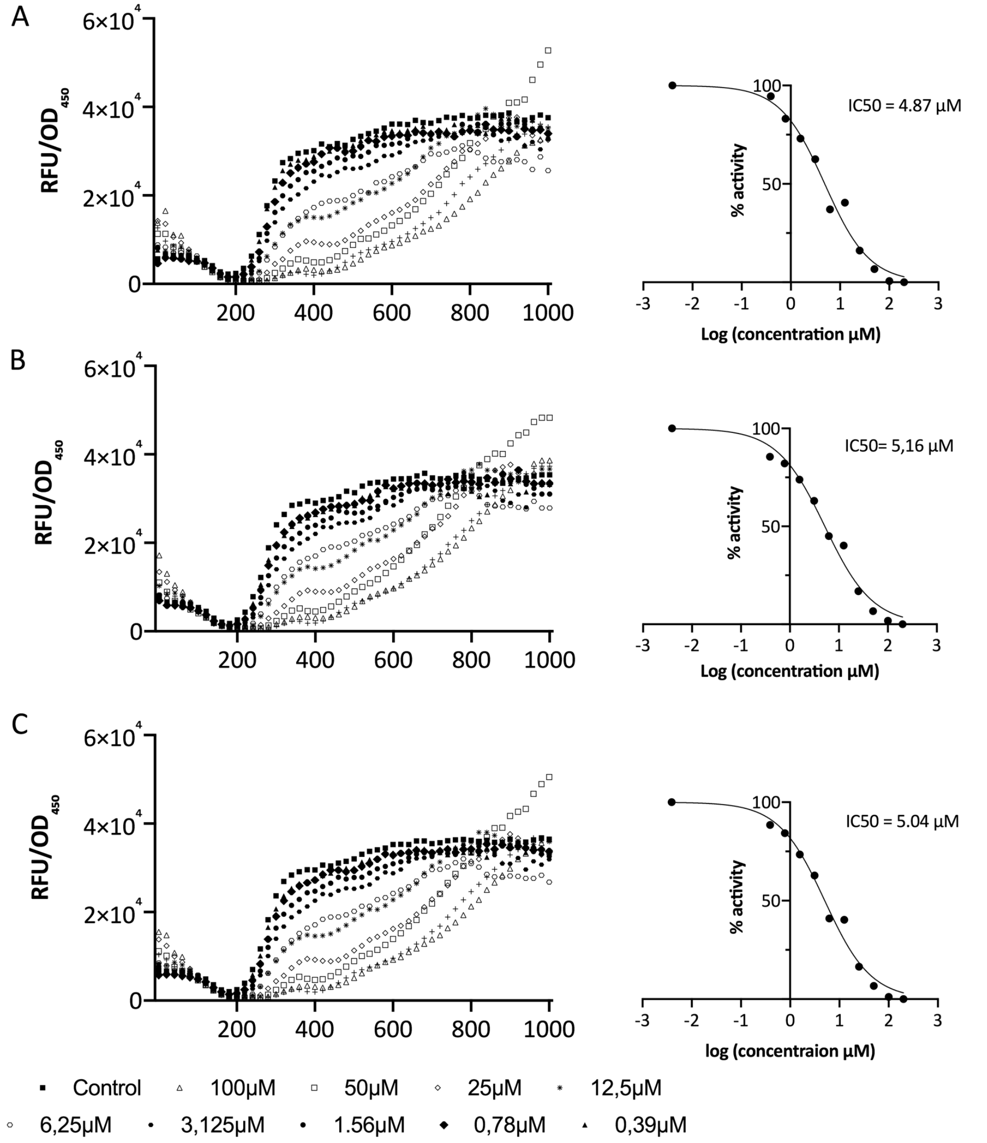

FIG S2 Inhibitory activity of Z/E-ethylthio enynone, Z-ethylthio enynone and Z/E-propylthio enynone on *rhlA* expression. Expression of *rhlA* measured as GFP intensity of a *rhlA-gfp* strain treated with 100 µM Z/E-ethylthio enynone (a), Z-ethylthio enynone (b) and Z/E-propylthio enynone (C). With corresponding curves for calculation of IC_{50} values.

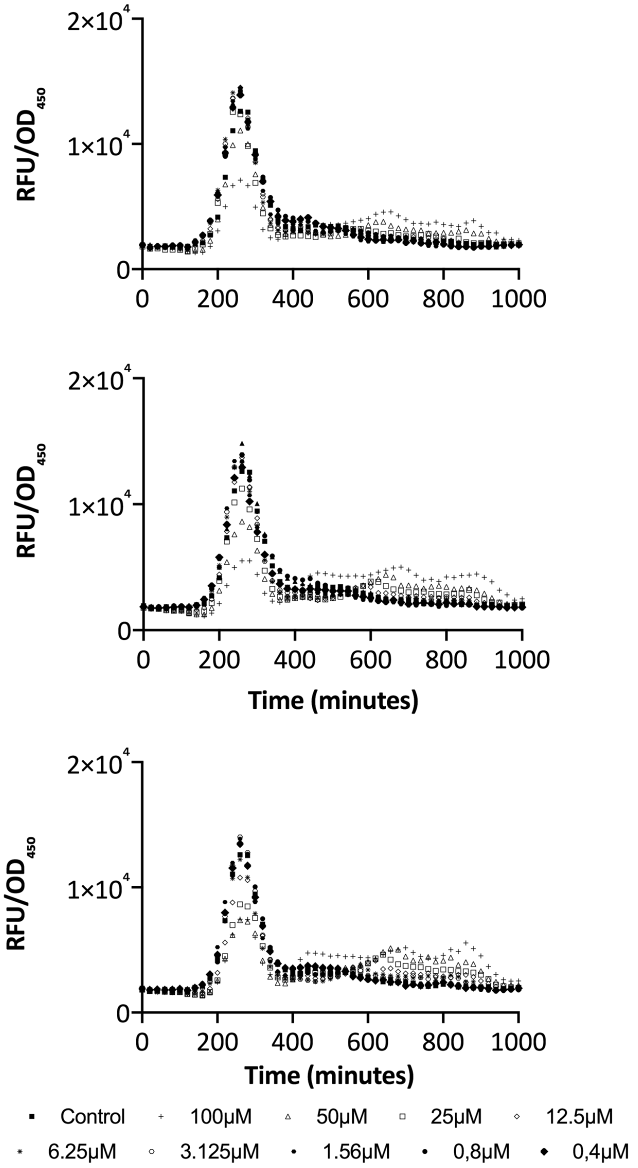

FIG S3 Inhibitory activity of Z/E-ethylthio enynone, Z-ethylthio enynone and Z/E-propylthio enynone on *pqsA* expression. Expression of *pqsA* measured as GFP intensity of a *pqsA-gfp* strain treated with 100 µM Z/E-ethylthio enynone (a), Z-ethylthio enynone (b) and Z/E-propylthio enynone (C).

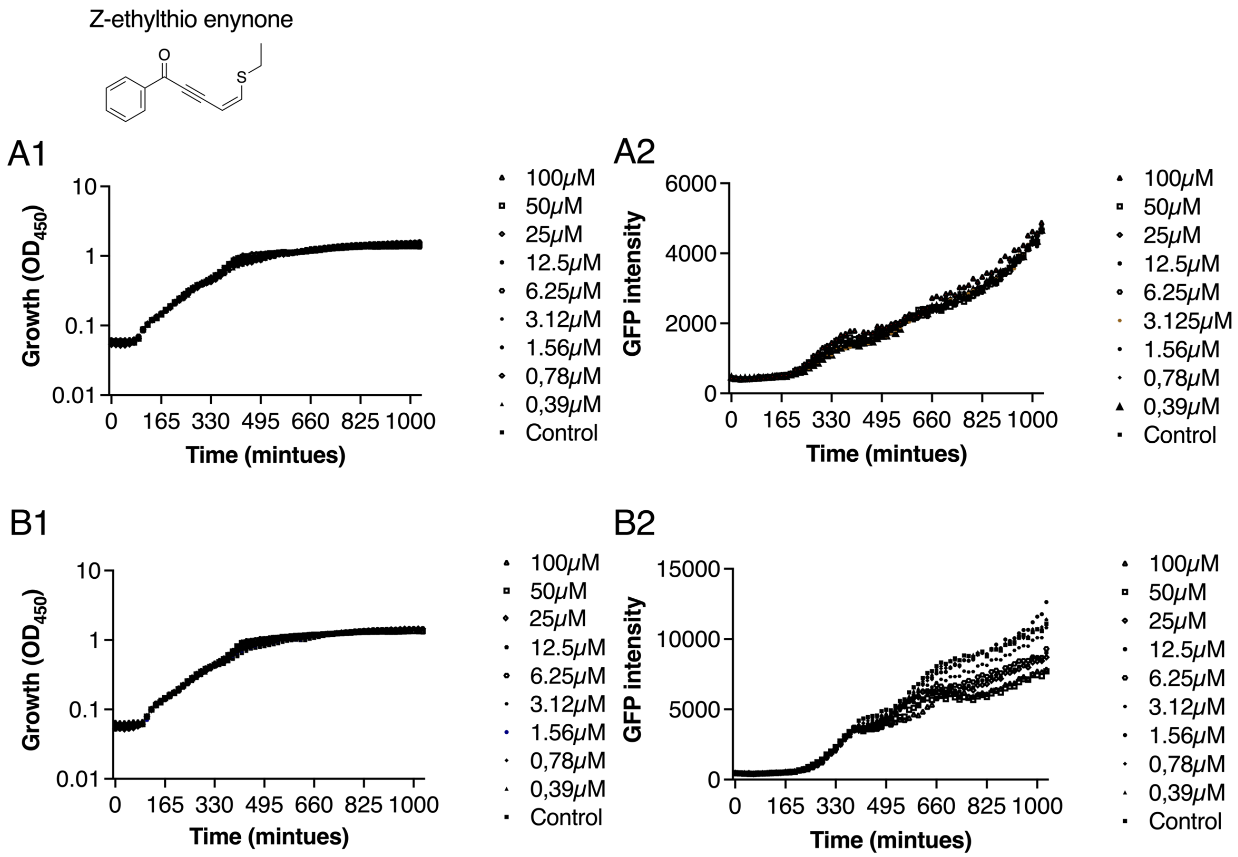

FIG S4 Inhibitory activity of Z-ethylthio enynone on *rsmY* and *rsmZ* expression. A1) Growth of *rsmY-gfp* monitor strain treated with 100 µM Z-ethylthio enynone. A2) Expression of *rsmY* measured as GFP intensity of a *rsmY-gfp* strain. B1) Growth of *rsmZ-gfp* monitor strain treated with 100 µM Z-ethylthio enynone. B2) Expression of *rsmZ* measured as GFP intensity of a *rsmZ-gfp* strain.

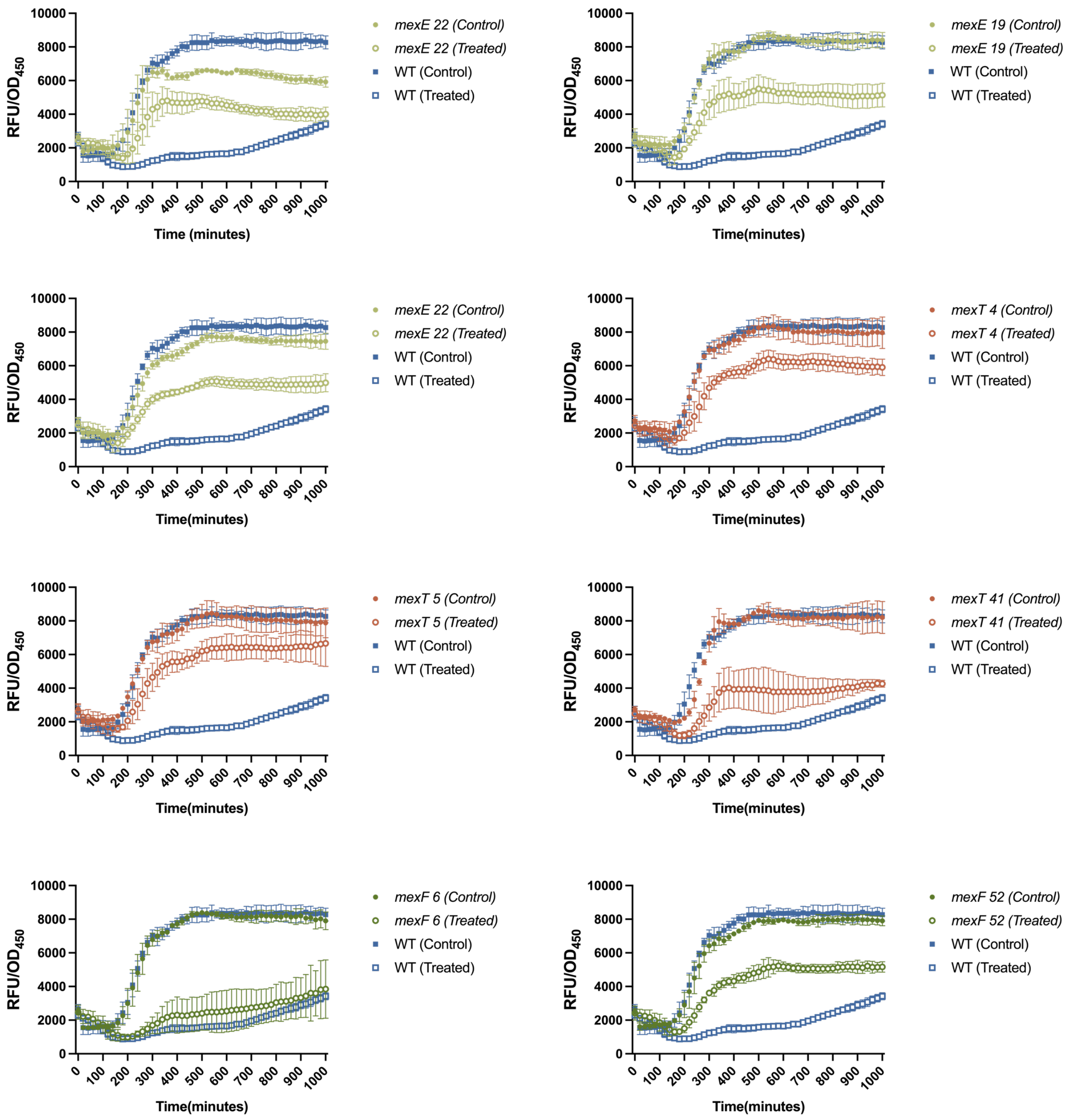

FIG S5 Effect of 100 µM Z-ethylthio enynone (Treated) on the fluorescence output of the CTX-*lasB-gfp* with transposon insertions in *mexT*, *mexE* and *mexF.* The transposons were isolated from the transposon mutagenesis experiment. WT: CTX-*lasB-gfp* monitor strain. The results are based on three independent experiments. Error bars represent means ± standard deviations (SD).

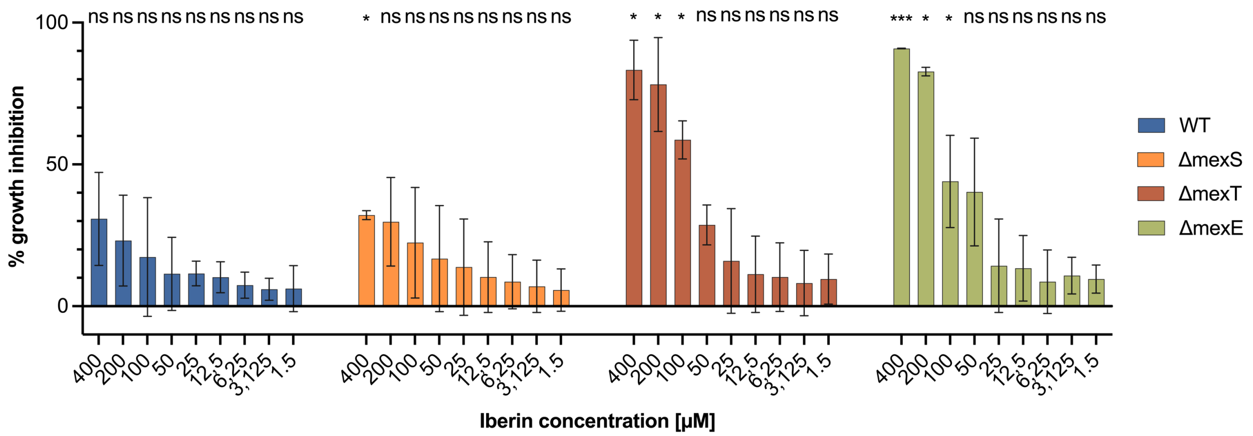

FIG S6 Effect of varying concentrations of iberin on the growth of the CTX-*lasB-gfp*, *mexS* mutant, *mexE* mutant and the *mexT* mutant. WT: CTX-*lasB-gfp* monitor strain. The results are based on three independent experiments. Error bars represent means ± standard deviations (SD). Significance levels from zero are based on one sample t-test analysis (ns P > 0.05, * P ≤ 0.05, ** p ≤ 0.01 and *** p ≤ 0.001).

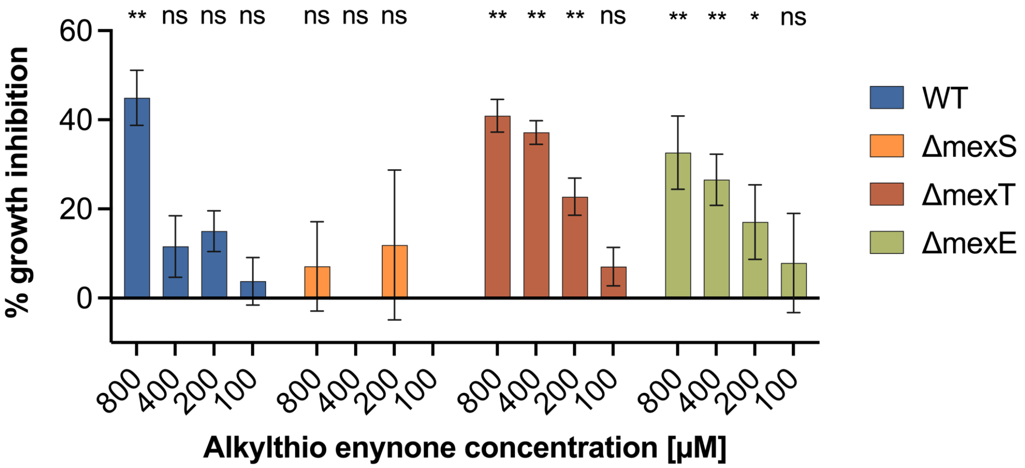

FIG S7 Effect of concentrations from 100-800 µM Z-ethylthio enynone on the growth of the CTX-*lasB-gfp*, *mexS* mutant, *mexE* mutant and *mexT* mutant. WT: CTX-*lasB-gfp* monitor strain. The results are based on three independent experiments. Error bars represent means ± standard deviations (SD). Significance levels from zero are based on one sample t-test analysis (ns P > 0.05, * P ≤ 0.05, ** p ≤ 0.01 and *** p ≤ 0.001).

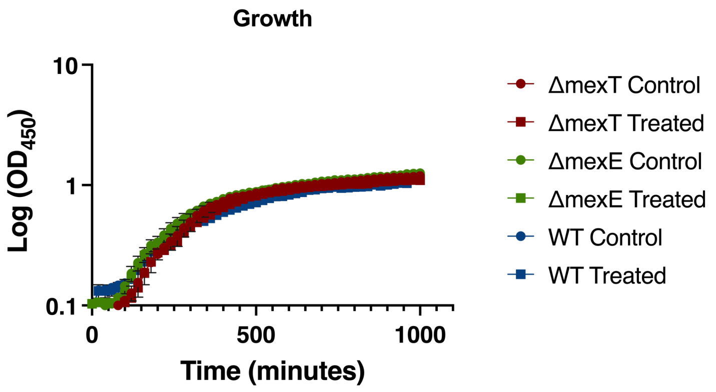

FIG S8 Growth curves of PAO1 WT and *mexE* and *mexT* mutants with and without treatment of 100 µM Z-ethylthio enynone. The results are based on three independent experiments. Error bars represent means ± standard deviations (SD).

Synthetic procedures

1,4-bis(trimethylsilyl)buta-1,3-diyne (**S1**)

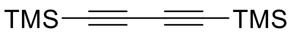

The Hay-coupling of two ethynyltrimethylsilane was achieved by mixing TMEDA (0.8 mL, 5.35 mmol, 0.21 eq.), CuI (423.4 mg, 2.2 mmol, 0.09 eq.) and DIPEA (13.5 mL, 77.5 mmol, 3 eq.) in THF (3.9 mL/mmol alkyne). To the mixture was then added ethynyltrimethylsilane (7.05 mL, 50.9 mmol, 2 eq.) and the reaction was allowed to stir overnight at 60°C. The following day the dark brown reaction mixture was confirmed complete by TLC, cooled to rt and filtered through Celite® with extra heptane. The product was purified on plug with pure heptane to obtain 3.29 g (67%) of the diyne **S1**. ^{1}H NMR (400 MHz, $CDCl_3$) δ 0.21 (s, 18H). ^{13}C NMR (101 MHz, $CDCl_3$) δ 87.97, 85.93, -0.51.

1-phenyl-5-(trimethylsilyl)penta-2,4-diyn-1-ol (**S2**)

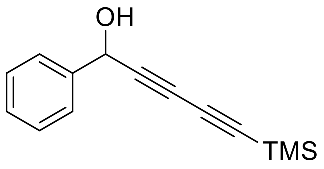

The bis(trimethylsilyl)butadiyne (1.01 g, 5.19 mmol, 1 eq.) was dissolved in dry THF (3.85 mL/mmol diyne) in a flame dried round-bottom flask under a N_2 atmosphere. The solution was cooled to 0°C and 1.50 M MeLi·LiBr in Et_2O (3.4 mL, 5.1 mmol, 1 eq.) was added under stirring. Afterwards the ice-bath was removed, and the reaction was allowed to stir for 4 hours. The mixture was then again cooled to 0°C and benzaldehyde (0.65 mL, 6.37 mmol, 1.2 eq.)

was added dropwise. After 1 hour on ice TLC confirmed the reaction complete. Sat. aq. NH4Cl was added and then the mixture was extracted with EtOAC, which was afterwards washed with Brine, dried over MgSO4, filtered and concentrated under reduced pressure. The residue was purified by flash chromatography EtOAc:heptane (10:90) to yield 958.1 mg (98%) of the secondary alcohol **S2** as a yellow oil. ^{1}H NMR (400 MHz, DMSO) δ 7.46 – 7.42 (m, 2H), 7.41 – 7.36 (m, 2H), 7.35 – 7.29 (m, 1H), 6.30 (d, *J* = 5.9 Hz, 1H), 5.50 (d, *J* = 6.0 Hz, 1H), 0.18 (s, 9H). ^{13}C NMR (101 MHz, DMSO) δ 141.37, 128.88, 128.39, 126.76, 88.26, 88.04, 80.81, 69.58, 63.16, -0.16.

1-phenyl-5-(trimethylsilyl)penta-2,4-diyn-1-one (**S3**)

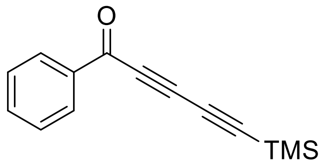

To a solution **S2** (2.5 g, 10.95 mmol, 1 eq.) in DCM (5.02 mL/mmol **S2**) was added MnO_2 (19.03 g, 218.95 mmol, 20 eq.). The reaction was refluxed under stirring for 2½ hours before TLC (20/80, EtOAc:heptane) showed completion. Then it was filtered through a silica plug, washed with additional DCM. The resulting solution was concentrated under reduced pressure to give 1.35 g (54%) of the diynone **S3**. ^{1}H NMR (400 MHz, DMSO) δ 8.10 – 8.01 (m, 2H), 7.82 – 7.72 (m, 1H), 7.61 (t, *J* = 7.8 Hz, 2H), 0.27 (s, 9H). ^{13}C NMR (101 MHz, DMSO) δ 176.40, 136.11, 135.73, 129.71 (x4), 98.60, 85.87, 76.39, 72.87, -0.53 (x3).

1-phenylpenta-2,4-diyn-1-one (**S4**)

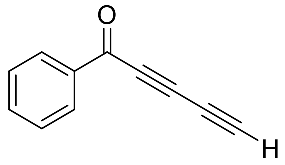

The diynone **S3** (57.4 mg, 0.25 mmol, 1 eq.) was dissolved in EtOAc (24 mL/mmol **S3**) and a premade mix of Na_2CO_3:Sillica (1:1, 10.8 g/mmol **S3**) was added. The suspension was allowed to stir for overnight or until TLC show full conversion (11 hours). The mixture was then purified by filtering through a silica plug. The free alkyne **S4** was used directly in the next step with assumed quant. yield. ^{1}H NMR (400 MHz, DMSO) δ 8.11 – 8.03 (m, 2H), 7.83 – 7.74 (m, 1H), 7.63 (t, *J* = 7.8 Hz, 2H), 4.62 (s, 1H). ^{13}C NMR (101 MHz, DMSO) δ 176.35, 136.03, 135.79, 129.73, 82.14, 76.37, 71.03, 66.24.

5-(ethylthio)-1-phenylpent-4-en-2-yn-1-one (**S6**)

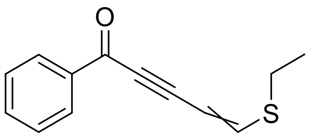

To a solution of the free alkyne (228 mg, 1.48 mmol, 1 eq.) in dry DCM (7 mL/mmol free alkyne) was added first EtSH (0.12 mL, 1.63 mmol, 1.1 eq.) to give a dark yellow solution, to which was added (n-Bu)$_3$P (37 µL, 0.15 mmol, 0.1 eq.). The mixture turns black instantly and TLC confirmed

reaction complete. The product was purified by plug (30:70, EtOAc:Heptane) to yield 262.8 mg (82%). The compound was further purified by preparative HPLC to give a single isomer (**S5**) as well as a mixture (**S6**). ^{1}H NMR (400 MHz, Chloroform-*d*) (**S5**) δ 8.28 – 8.24 (m, 2H), 7.64 – 7.58 (m, 1H), 7.51 (dd, *J* = 8.5, 7.0 Hz, 2H), 7.06 (d, *J* = 10.0 Hz, 1H), 5.75 (d, *J* = 10.0 Hz, 1H), 2.92 (q, *J* = 7.4 Hz, 2H), 1.41 (t, *J* = 7.4 Hz, 3H). ^{13}C NMR (101 MHz, Chloroform-*d*) (**S5**) δ 177.82, 149.70, 136.95, 133.89, 129.73, 128.56, 101.95, 94.90, 90.16, 28.40, 15.77.

1-phenyl-5-(propylthio)pent-4-en-2-yn-1-one (**S7**)

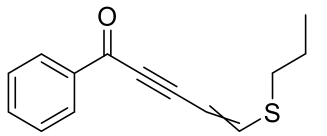

To a solution of the free alkyne (145.55 mg, 0.94 mmol, 1 eq.) in dry DCM (7 mL/mmol free alkyne) was added first PrSH (97 µL, 1.05 mmol, 1.1 eq.) and afterwards (n-Bu)$_3$P (24 µL, 0.94 mmol, 0.1 eq.). The reaction was complete instantly which TLC confirmed. The product was purified by plug (10:90, EtOAc:Heptane) to yield 182.3 mg (84%) and afterwards further purified on preparative HPLC to give the isomeric mixture of **S7**. ^{1}H NMR (400 MHz, Chloroform-*d*) δ 8.30 – 8.24 (m, 2H), 7.65 – 7.59 (m, 1H), 7.55 – 7.48 (m, 2H), 7.05 (d, *J* = 10.0 Hz, 1H), 5.73 (d, *J* = 10.0 Hz, 1H), 2.89 (t, *J* = 7.3 Hz, 2H), 1.76 (tq, *J* = 7.2 Hz, 2H), 1.07 (d, *J* = 7.4 Hz, 3H). ^{13}C NMR (101 MHz, Chloroform-*d*) δ 176.20, 150.21, 136.95, 133.86, 129.75, 128.54, 116.44, 101.74, 90.22, 36.40, 23.89, 13.03.

Supplemental references

1. Stover CK, Pham XQ, Erwin AL, Mizoguchi SD, Warrener P, Hickey MJ, et al. Complete genome sequence of Pseudomonas aeruginosa PAO1, an opportunistic pathogen. Nature. 2000;406(6799):959-64; doi: 10.1038/35023079.
2. Jakobsen TH, Warming AN, Vejborg RM, Moscoso JA, Stegger M, Lorenzen F, et al. A broad range quorum sensing inhibitor working through sRNA inhibition. Sci Rep. 2017;7(1):9857; doi: 10.1038/s41598-017-09886-8.
3. Pamp SJ, Tolker-Nielsen T. Multiple roles of biosurfactants in structural biofilm development by Pseudomonas aeruginosa. Journal of bacteriology. 2007;189(6):2531-9; doi: 10.1128/JB.01515-06.
4. Skindersoe ME, Alhede M, Phipps R, Yang L, Jensen PO, Rasmussen TB, et al. Effects of antibiotics on quorum sensing in Pseudomonas aeruginosa. Antimicrob Agents Chemother. 2008;52(10):3648-63; doi: 10.1128/AAC.01230-07.
5. Hmelo LR, Borlee BR, Almblad H, Love ME, Randall TE, Tseng BS, et al. Precision-engineering the Pseudomonas aeruginosa genome with two-step allelic exchange. Nat Protoc. 2015;10(11):1820-41; doi: 10.1038/nprot.2015.115.
6. Hoang TT, Kutchma AJ, Becher A, Schweizer HP. Integration-proficient plasmids for Pseudomonas aeruginosa: site-specific integration and use for engineering of reporter and expression strains. Plasmid. 2000;43(1):59-72; doi: 10.1006/plas.1999.1441.
7. Kulasekara HD, Ventre I, Kulasekara BR, Lazdunski A, Filloux A, Lory S. A novel two-component system controls the expression of Pseudomonas aeruginosa fimbrial cup genes. Mol Microbiol. 2005;55(2):368-80; doi: 10.1111/j.1365-2958.2004.04402.x.
8. Jakobsen TH, van Gennip M, Phipps RK, Shanmugham MS, Christensen LD, Alhede M, et al. Ajoene, a Sulfur-Rich Molecule from Garlic, Inhibits Genes Controlled by Quorum Sensing. Antimicrob Agents Ch. 2012;56(5):2314-25; doi: 10.1128/AAC.05919-11.
9. Hentzer M, Wu H, Andersen JB, Riedel K, Rasmussen TB, Bagge N, et al. Attenuation of Pseudomonas aeruginosa virulence by quorum sensing inhibitors. The EMBO journal. 2003;22(15):3803-15; doi: 10.1093/emboj/cdg366.
10. Fetar H, Gilmour C, Klinoski R, Daigle DM, Dean CR, Poole K. mexEF-oprN multidrug efflux operon of Pseudomonas aeruginosa: regulation by the MexT activator in response to nitrosative stress and chloramphenicol. Antimicrob Agents Chemother. 2011;55(2):508-14; doi: 10.1128/AAC.00830-10.
11. Jakobsen TH, Bragason SK, Phipps RK, Christensen LD, van Gennip M, Alhede M, et al. Food as a Source for Quorum Sensing Inhibitors: Iberin from Horseradish Revealed as a Quorum Sensing Inhibitor of Pseudomonas aeruginosa. Appl Environ Microb. 2012;78(7):2410-21; doi: 10.1128/AEM.05992-11.
